# Racemic ketamine in adult head injury patients: use in endotracheal suctioning

**DOI:** 10.1186/cc13097

**Published:** 2013-11-08

**Authors:** Anselmo Caricato, Alessandra Tersali, Sara Pitoni, Chiara De Waure, Claudio Sandroni, Maria Grazia Bocci, Maria Giuseppina Annetta, Mariano Alberto Pennisi, Massimo Antonelli

**Affiliations:** 1 Institute of Anesthesiology and Intensive Care, Catholic University School of Medicine, Policlinico ‘A. Gemelli’, Rome, Italy; 2 Institute of Hygiene, Catholic University School of Medicine, Policlinico ‘A. Gemelli’, Rome, Italy

## Abstract

**Introduction:**

Endotracheal suctioning (ETS) is essential for patient care in an ICU but may represent a cause of cerebral secondary injury. Ketamine has been historically contraindicated for its use in head injury patients, since an increase of intracranial pressure (ICP) was reported; nevertheless, its use was recently suggested in neurosurgical patients. In this prospective observational study we investigated the effect of ETS on ICP, cerebral perfusion pressure (CPP), jugular oxygen saturation (SjO_2_) and cerebral blood flow velocity (mVMCA) before and after the administration of ketamine.

**Methods:**

In the control phase, ETS was performed on patients sedated with propofol and remifentanil in continuous infusion. If a cough was present, patients were assigned to the intervention phase, and 100 γ/kg/min of racemic ketamine for 10 minutes was added before ETS.

**Results:**

In the control group ETS stimulated the cough reflex, with a median cough score of 2 (interquartile range (IQR) 1 to 2). Furthermore, it caused an increase in mean arterial pressure (MAP) (from 89.0 ± 11.6 to 96.4 ± 13.1 mmHg; *P* <0.001), ICP (from 11.0 ± 6.7 to 18.5 ± 8.9 mmHg; *P* <0.001), SjO_2_ (from 82.3 ± 7.5 to 89.1 ± 5.4; *P* = 0.01) and mVMCA (from 76.8 ± 20.4 to 90.2 ± 30.2 cm/sec; *P* = 0.04). CPP did not vary with ETS. In the intervention group, no significant variation of MAP, CPP, mVMCA, and SjO_2_ were observed in any step; after ETS, ICP increased if compared with baseline (15.1 ± 9.4 vs. 11.0 ± 6.4 mmHg; *P* <0.05). Cough score was significantly reduced in comparison with controls (*P* <0.0001).

**Conclusions:**

Ketamine did not induce any significant variation in cerebral and systemic parameters. After ETS, it maintained cerebral hemodynamics without changes in CPP, mVMCA and SjO_2_, and prevented cough reflex. Nevertheless, ketamine was not completely effective when used to control ICP increase after administration of 100 γ/kg/min for 10 minutes.

## Introduction

Endotracheal suctioning (ETS) is essential for patient care in an intensive care unit, but may represent a cause of cerebral secondary injury. In the last few years, several authors found that it can have considerable negative impact on cerebral physiologic variables, causing coughing, increasing intracranial pressure (ICP) and altering cerebral perfusion pressure (CPP) in head-injured patients [1-5].

With the aim of controlling cerebral and systemic effects related to ETS, a lot of studies have been conducted to test different drugs or procedures, such as hyperventilation [6], administration of opioids [7-10], barbiturates [8], local anesthetics [8,11-13], and paralyzing agents [7,8]. Unfortunately, in many cases single-center nonrandomized clinical studies were performed, based on small samples, thus leading to conclusions with low level of scientific evidence. Even if recent recommendation for ETS in mechanically ventilated patients are available [14], so far no consensus exists about which strategy should be used to reduce intracranial effects of this procedure.

Ketamine is an N-methyl-D-aspartate receptor antagonist recommended during minor procedures, for the induction of anesthesia prior to the administration of other agents, and to supplement low-potency anesthetics. It has unique properties as a dissociative anesthetic, analgesic, amnestic and anxiolytic; it exerts its effect by ‘disconnecting’ the thalamocortical and limbic systems, effectively dissociating the central nervous system from outside stimuli [15,16].

The result is a ‘dissociative anesthesia’, that is characterized by analgesia, amnesia and anxiolysis, while maintaining cardiovascular stability and preserving spontaneous respirations and protective airway reflexes [15,16].

It has been historically contraindicated for its use in patients with head injury because of a concern that it may increase ICP [17-21]. However, several recent studies suggested ketamine as a sedative in the setting of a neurosurgical intensive care unit, since no association was observed between ketamine and ICP in mechanically ventilated head-injured patients during continuous analgosedation [22-29].

Thus, it has officially been recommended for use in analgesia and sedation in European countries.

Furthermore, several animal studies observed its neuroprotective activity against hypoxic, ischemic or mechanical neuronal insults [29].

We hypothesized that ketamine could be safe and effective in blunting ICP increase after ETS in these patients.

Thus, we investigated the effect of ETS before and after ketamine on ICP, CPP, jugular oxygen saturation (SjO_2_) and cerebral blood flow velocity in head-injured patients during continuous infusion of propofol and remifentanil.

## Material and methods

### Patients and data collection

After approval by the Ethics Committee of the Catholic University School of Medicine (P/997/CE/2010), informed written consent was obtained from patients’ next of kin.

Inclusion criteria were:

● Age between 18 and 75 years

● Severe closed head-injured patients (GCS ≤8) within 72 hours after trauma

● ICP monitoring

● Analgesia and sedation with propofol (3 to 5 mg/kg/h) and remifentanil (0.05 to 2 γ/kg/min) in continuous infusion to obtain a Ramsey score of 5 to 6

Exclusion criteria were:

● Severe hemodynamic instability

● Paralyzed patients

● Known allergy to the treatment drugs or pregnancy

Patients were managed according to Brain Trauma Foundation Guidelines [30]. Multimodal monitoring, including ICP and SjO_2_ was performed (SC7000 Monitor, Siemens, Erlangen, Germany). Patients were mechanically ventilated in volume-controlled ventilation, thus to maintain partial pressure of carbon dioxide in the blood (paCO_2_) between 32 and 35 mmHg and partial pressure of oxygen in the blood (paO_2_) >70 mmHg.

Jugular bulb catheters were inserted in the larger internal jugular vein, under ultrasonographic control; correct position was verified by X-rays.

Patients were nursed supine with a 30° head-up tilt. Blood flow velocity in the middle cerebral artery (mV MCA) was measured by 2 MHz pulsed Doppler ultrasound device (transcranial Doppler (TCD) H21-Hitachi Medical Systems Europe, Zug, Switzerland).

### Study protocol

The study was structured in two phases: control and intervention. In the control phase, patients were sedated with propofol (3 to 5 mg/kg/h) and remifentanil (0.05 to 0.2 γ/kg/min) in continuous infusion, and ETS was performed according to recent guidelines through an orotracheal tube [14]. If cough was present, patients were assigned to the intervention phase, and an infusion of a racemic mixture of ketamine 100 γ/kg/min for 10 minutes was added before ETS.

In both phases, each patient underwent the protocol as described in Table 1. At each step, a standard set of parameters was recorded: heart rate (HR), mean arterial pressure (MAP), ICP, CPP. In addition, at step 1, 3 and 5, arterial blood gas analysis and SjO_2_ were carried out; at these steps, mV MCA was bilaterally measured by TCD, and mean value was reported. In the control phase, step 2 was not carried out.

**Table 1:** Study protocol

**Study protocol**	
T1	Baseline
T2	Immediately before ETS
T3	Immediately after ETS
T4	5 minutes after ETS
T5	10 minutes after ETS

An evaluation of cough strength was performed after ETS by a semi-quantitative scale (Harris Scale; component ‘Response to endotracheal suctioning’) [31]. It ranged between 1 and 4 (Table 2).

**Table 2:** **Scoring of cough strength**[31]

**Cough strength**	
1	Agitation, distress, prolonged coughing
2	Coughs, distressed, rapid recovery
3	Coughs, not distressed
4	No cough

### Statistical analysis

Repeated measures analysis of variance was used in order to study physiologic parameters before, during and after ETS. Cough reflex after ETS was evaluated by the Friedman test for repeated measures; in cases of multiple comparisons, level of significance was adjusted using the Bonferroni correction, and corrected *P* value was reported. Statistical calculations were performed using the Statistical Package for Social Sciences (Windows version 14.0, Microsoft Corp, Redwood, WA, USA). Results are reported as mean ± standard deviation, or median and interquartile range (IQR), as appropriated; a value of *P* <0.05 was considered statistically significant.

## Results

Twenty-one head-injured patients consecutively admitted to 18-bed general ICU of ‘A. Gemelli’ Hospital between 1 January 2011 and 1 November 2011 were enrolled in the study. ‘A. Gemelli’ Hospital is a 1,200-bed university hospital located in Rome, Italy, that is a referral center for severely injured patients and serves an urban area of 1 million people.

During baseline analgosedation (control group), a cough reflex was present in all patients, and all were included in the study.

The sample was predominantly male (57%), with nine females (43%). The mean age was 54.6 ± 21.2, ranging from 19 to 74 yrs. The patients’ severity of illness, as measured by the Glasgow Coma Scale (5.8 ± 3.8) and the Simplified Acute Physiology Score II (SAPS II) score (49.7 ± 9.2), showed severe injuries and significant comorbidities requiring high levels of medical and nursing care. At baseline, ICP was always below 25 mmHg. During the study, hyperventilation was not applied, and five patients were treated with hyperosmolar therapy.

### Control

**Figure 1:**
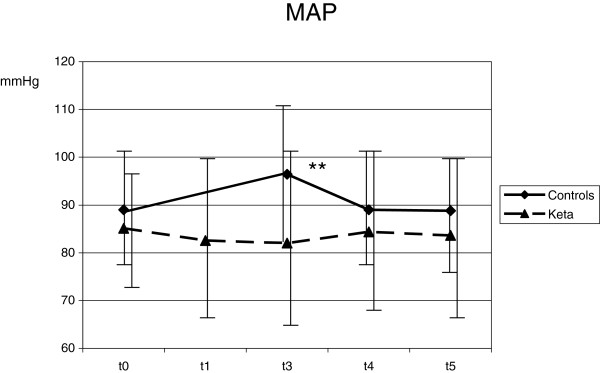
**Ketamine prevented any significant increase of mean arterial pressure (MAP) after endotracheal suctioning.** Statistical significance in comparison with T1 is shown. *P <0.05; **P <0.01.

**Figure 2:**
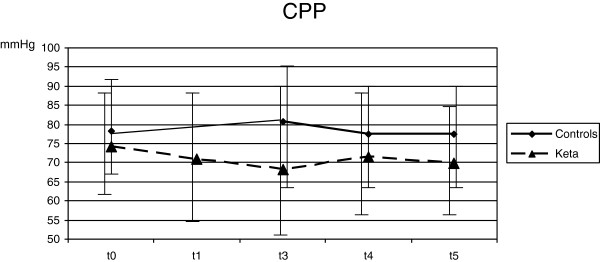
No significant difference in cerebral perfusion pressure (CPP) was observed during the study in both groups.

**Figure 3:**
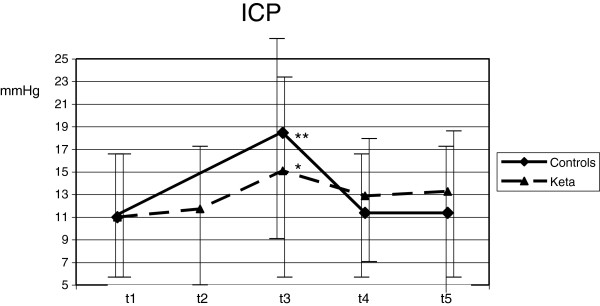
**Differences between control and intervention in intracranial pressure (ICP) during the study are shown.** Statistical significance in comparison with T1 is shown. *P <0.05; **P <0.01.

**Figure 4:**
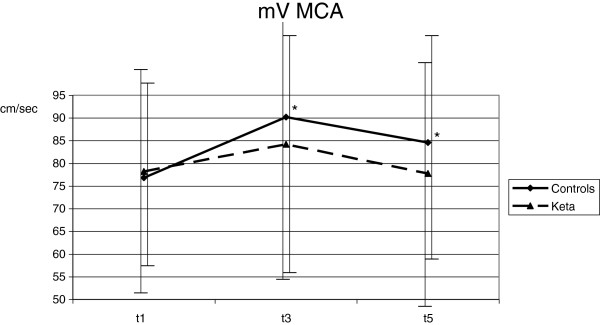
**A significant increase of blood flow velocity in the middle cerebral artery (mV MCA) was observed in the control group.** Statistical significance in comparison with T1 is shown. *P <0.05.

**Figure 5:**
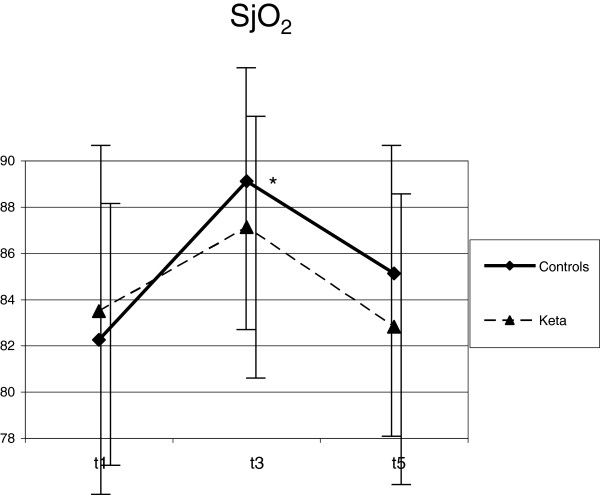
**SjO2 increased after endotracheal suctioning (ETS) in both groups.** In the control group a significant difference was observed. Statistical significance in comparison with T1 is shown. *P <0.05.

**Figure 6:**
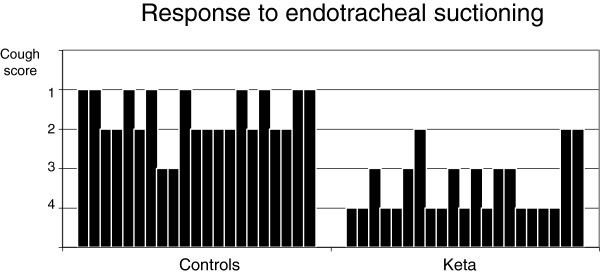
**Cough score evaluated after endotracheal suctioning (ETS) is shown **[31]**.** Each bar corresponds to the cough score of each patient.

Despite deep sedation, ETS (T3) caused an increase of HR (from 74.3 ± 14.0 to 82.1 ± 17.6 min^-1^; *P* <0.001), MAP (from 89.0 ± 11.6 to 96.4 ± 13.1 mmHg; *P* <0.001), ICP (from 11.0 ± 6.7 to 18.5 ± 8.9 mmHg; *P* <0.001), SjO_2_ (from 82.3 ± 7.5 to 89.1 ± 5.4%; *P* = 0.01) and mV MCA (from 76.8 ± 20.4 to 90.2 ± 30.2 cm/sec; *P* =0.04) if compared with T1. CPP did not vary with ETS (Figures 1, 2, 3, 4 and 5). Furthermore, ETS stimulated a strong cough reflex, with a median cough score of 2 (IQR 1 to 2) (Figure 6).

These changes were not sustained over time. As compared with T1, 10 minutes after ETS (T5) HR (from 74.3 ± 14.0 to 78.0 ± 20.4 min^-1^; *P* = 0.17), MAP (from 89.0 ± 11.6 to 88.8 ± 11.0 mmHg; *P* = 0.92), ICP (from 11.0 ± 6.7 to 11.4 ± 7.4 mmHg; *P* = 0.61) and SjO2 (from 82.3 ± 7.5 to 85.1 ± 6.0%; *P* = 0.13) were not modified; mV MCA increased (from 76.8 ± 20.4 to 84.6 ± 24.2; *P* = 0.03) (Figures 1, 2, 3, 4 and 5).

### Intervention

No significant variation of HR, MAP, CPP, mV MCA, and SjO_2_ were observed in any step during the study. At baseline (T1) ICP was 11.0 ± 6.4 mmHg; at this step, in two cases it was higher than 20 mmHg. Following ketamine administration (T2), ICP did not modify (11.7 ± 7.3 vs. 11.0 ± 6.4 mmHg; *P* = 0.28); after ETS (T3) cough reflex was significantly reduced in comparison with controls (cough score 4 (IQR 3 to 4) vs. 2 (IQR1 to 2); *P* <0.0001) (Figure 6). ICP increased after ETS if compared with T1 (15.1 ± 9.4 vs. 11.0 ± 6.4 mmHg; *P* <0.05). (Figures 1, 2, 3, 4 and 5). Interestingly, in contrast with controls, ETS did not induce any significant change of mV MCA and SjO_2_.

## Discussion

The main result of this study is that the administration of a racemic mixture of ketamine did not induce any significant variation of ICP, CPP, MAP, mV MCA and SjO_2_ in mechanically ventilated head-injured patients during continuous analgosedation. If administered before ETS, racemic ketamine reduced cough reflex, and prevented any significant change of MAP, CPP, mV MCA and SjO_2_ in comparison with controls. Nevertheless, its use before ETS was not sufficient to completely blunt ICP increases at the drug dose studied.

Ketamine has been historically contraindicated for its use in patients with head injury, since an association with increased ICP was reported [17-21]. This concept originated from a few case reports and small case–control studies from the 1970s conducted on patients with abnormal cerebrospinal fluid pathways. The increase of ICP was observed in patients who were breathing spontaneously and who had received ketamine as a sole anesthetic agent. It was thought to be related to an increase in cerebral metabolic rate and to a corresponding increase of cerebral blood flow.

However, several articles are challenging this message [22-29]. Mayberg *et al.* investigated cerebral hemodynamics in 20 patients undergoing craniotomy after induction of isoflurane/nitrous oxide anesthesia [23]. They found that an intravenous bolus of 1 mg/kg ketamine did not modify MAP, CPP and arterojugular difference of oxygen, while ICP and mV MCA were significantly reduced. Albanese *et al.* confirmed these data [24]. In patients with severe head injury who were sedated with propofol, they found that ICP decreased after increasing doses of intravenous ketamine boluses, and no significant differences in MAP, CPP, SjO_2_, and mV MCA were observed. Recently, Bar-Joseph found that a single ketamine dose decreased ICP by 30% (from 25.8 ± 8.4 to 18.0 ± 8.5 mm Hg; *P* <0.001) and increased CPP from 54.4 ± 11.7 to 58.3 ± 13.4 mm Hg (*P* <0.005) during analgosedation in pediatric mechanically ventilated head-injured patients [22].

Reasons for these conflicting results are not completely known. In particular, effects of ketamine on cerebral blood flow (CBF) and cerebral metabolic rate (CMR) are equivocal, since they varied in different brain regions, according to the type of ketamine used (racemic, S-, or R- enantiomers) and the dose administered. According to positron emission tomography (PET) studies by Vollenweider, subanesthetic doses (0.2 to 0.3 mg/kg) of S-ketamine increased CMR, whereas R-ketamine decreased it [32]. Schmidt demonstrated a dramatic decrease in CBF following the administration of a large (10 mg/kg) dose of racemic ketamine [33].

Also, the observed increase in CBF may be partly mediated by a direct effect of the drug on arterial pressure, and partly by a concomitant increase in PaCO_2_ in spontaneously breathing patients.

Simultaneous administration of propofol or benzodiazepines, and mechanical ventilation may blunt these changes in CBF, and explain the results of recent studies.

At present, the use of ketamine in neurosurgical patients is not considered completely safe. Even if it is recommended in some countries for analgesia and sedation in head-injured patients, the Federal Drug Administration (FDA) suggests its use with extreme caution in patients with preanesthetic elevated cerebrospinal fluid pressure. The Italian Authority for Drugs (AIFA) contraindicate its use in patients with head injury [34]. Recent guidelines on dealing with sedation and anesthesia for traumatic brain injury do not mention it [30].

Our work provides support not only for the absence of any significant variation of ICP after ketamine, but also for the stability of mV MCA and SjO_2_. In addition, we observed that the sedative effects of ketamine might be useful as an adjunct to continuous analgosedation for blunting cerebral and systemic response after ETS.

Intracranial effects of ETS have been extensively studied.

Several authors suggested that vasodilation occurs during ETS, with a resulting increase in CBF that is partially responsible for the increase in ICP [2,35,36]. Cruz found that ETS increased MAP and ICP, with a concomitant increase in SjO_2_ and mV MCA, suggesting a systemic and cerebral response to painful stimulus [37]. In addition, coughing may induce an increase of intrathoracic pressure and central venous pressure that may contribute to ICP elevation [38].

Our data confirm these results. Despite analgosedation, ETS caused an increase of MAP associated with an elevation of mV MCA and SjO_2_, as in the presence of an increase of CBF. ICP significantly increased, but came back to baseline after 10 minutes. This transient increase in ICP after ETS may be due to the fact that intracranial hypertension was not common in our patients. Actually, mV MCA was significantly increased, and it remained high during all the study. Unfortunately, we did not measure CBF, but it is reasonable that in patients with an altered intracranial compliance, the increase of CBF may induce a severe increase of ICP.

After ketamine, cough reflex was significantly reduced with respect to controls. In the same way, we observed only an increase of ICP, in absence of any significant variation of systemic and cerebral parameters. During ICP increase, MAP did not modify, and CPP showed a slight and nonsignificant reduction. In contrast with what we observed in controls, we did not find any significant variation of mV MCA and SjO_2_, suggesting that ketamine could have prevented the increase of CBF induced by ETS.

These data were in accord with Bar-Joseph, who observed that ketamine reduced ICP in 88% of cases during a potentially distressing intervention such as respiratory physiotherapy, endotracheal suctioning or bed linen change [22]. They did not study a standardized noxious stimulus, as we did, and probably cerebral effects were related to a less painful procedure.

Nevertheless, their results refute the notion that ketamine could increase ICP, suggesting that ketamine could induce an additional anesthetic effect without decrease of CPP.

We are aware that this study has some limitations. In fact, it was observational, monocentric and conducted on a small number of patients. Furthermore intracranial hypertension was not common in our cases and we did not know in how many cases autoregulation was impaired. Probably, cerebral effects of ETS after ketamine could be different in those subset of patients.

We used ketamine at a dose of 100 γ/kg/min for 10 minutes before ETS to minimize hemodynamic variation due to rapid infusion. Actually, this dose can be low for clinical effects; moreover, because of its brief half-life, this slow infusion rate may lead to a less effective action of the drug.

## Conclusions

In conclusion, our data showed that racemic ketamine did not induce any significant variation in cerebral and systemic parameters in mechanically ventilated head-injured patients during continuous analgosedation. After ETS, ketamine maintained cerebral hemodynamics without changes in CPP, mV MCA and SjO_2_, and prevented cough reflex. Nevertheless, it was not effective enough to control ICP increase.

Further studies should be encouraged to confirm these results, in order to update indications of ketamine in neurosurgical patients, and to recommend the best strategy to control cerebrovascular effects after ETS.

## Key messages

● Ketamine did not induce any significant variation of ICP, CPP, MAP, mV MCA and SjO_2_ in mechanically ventilated head-injured patients during continuous analgosedation

● Despite continuous infusion of analgosedation, endotracheal suctioning stimulated cough reflex and caused an increase of MAP, SjO2, mV MCA and ICP.

● If added to continuous analgosedation before endotracheal suctioning, ketamine reduced cough reflex, and prevented any significant change of MAP, CPP, mV MCA and SjO_2_

● Ketamine was not sufficient to completely blunt ICP increases after endotracheal suctioning.

## Abbreviations

AIFA: Italian authority for drugs; CBF: Cerebral blood flow; CMR: Cerebral metabolic rate; CPP: Cerebral perfusion pressure; ETS: Endotracheal suctioning; FDA: Federal Drug Administration; GCS: Glasgow coma scale; HR: Heart rate; ICP: Intracranial pressure; ICU: Intensive care unit; IQR: Interquartile range; MAP: Mean arterial pressure; mV MCA: Mean velocity in the middle cerebral artery; PET: Positron emission tomography; SAPS II: Simplified acute physiology score II; SjO2: Jugular oxygen saturation.

## Competing interests

The authors declare that they have no competing interests.

## Authors’ contributions

AC had full access to all the data in the study and takes responsibility for the integrity and the accuracy of the data analysis. AC conceived the study, and participated in its design and coordination and helped to draft the manuscript. SP participated in analysis and interpretation of data, helped to draft the manuscript, and critically revised the manuscript for important intellectual content. SP, AT, and MGB collected the data for the study and participated in statistically analysis. CS, MGA, MAP and MA participated in the conception, design and development of the database, helped in analysis and interpretation of data, helped in drafting of the manuscript and critically revised the manuscript for important intellectual content. CDW was in charge of the statistical analysis, and critically revised the manuscript. All authors read and approved the final manuscript.
